# The Patient Health Questionnaire-9 for detection of major depressive disorder in primary care: consequences of current thresholds in a crosssectional study

**DOI:** 10.1186/1471-2296-11-98

**Published:** 2010-12-13

**Authors:** Nicolaas PA Zuithoff, Yvonne Vergouwe, Michael King, Irwin Nazareth, Manja J van Wezep, Karel GM Moons, Mirjam I Geerlings

**Affiliations:** 1 University Medical Center Utrecht, Julius Center for Health Sciences and Primary Care, the Netherlands; 2 Department of Mental Health Sciences, Royal Free and University College Medical School, UK; 3 Netherlands Institute of Mental Health and Addiction, Trimbos Institute, Utrecht, the Netherlands

## Abstract

**Background:**

There is a need for brief instruments to ascertain the diagnosis of major depressive disorder. In this study, we present the reliability, construct validity and accuracy of the PHQ-9 and PHQ-2 to detect major depressive disorder in primary care.

**Methods:**

Cross-sectional analyses within a large prospective cohort study (PREDICT-NL). Data was collected in seven large general practices in the centre of the Netherlands. 1338 subjects were recruited in the general practice waiting room, irrespective of their presenting complaint. The diagnostic accuracy (the area under the ROC curve and sensitivities and specificities for various thresholds) was calculated against a diagnosis of major depressive disorder determined with the Composite International Diagnostic Interview (CIDI).

**Results:**

The PHQ-9 showed a high degree of internal consistency (ICC = 0.88) and test-retest reliability (correlation = 0.94). With respect to construct validity, it showed a clear association with functional status measurements, sick days and number of consultations. The discriminative ability was good for the PHQ-9 (area under the ROC curve = 0.87, 95% CI: 0.84-0.90) and the PHQ-2 (ROC area = 0.83, 95% CI 0.80-0.87). Sensitivities at the recommended thresholds were 0.49 for the PHQ-9 at a score of 10 and 0.28 for a categorical algorithm. Adjustment of the threshold and the algorithm improved sensitivities to 0.82 and 0.84 respectively but the specificity decreased from 0.95 to 0.82 (threshold) and from 0.98 to 0.81 (algorithm). Similar results were found for the PHQ-2: the recommended threshold of 3 had a sensitivity of 0.42 and lowering the threshold resulted in an improved sensitivity of 0.81.

**Conclusion:**

The PHQ-9 and the PHQ-2 are useful instruments to detect major depressive disorder in primary care, provided a high score is followed by an additional diagnostic work-up. However, often recommended thresholds for the PHQ-9 and the PHQ-2 resulted in many undetected major depressive disorders.

## Background

Assessment of major depressive disorder with (semi-)structured interviews such as the CIDI or the SCID[1,2], can be time consuming in the primary care setting. There is a need for using brief instruments such as the Patient Health Questionnaire-9 (PHQ-9) and the PHQ-2[3], to ascertain the diagnosis of major depressive disorder.

The PHQ-9 is derived from PRIME-MD[4] which was originally developed to detect five common mental disorders in primary care: depression, anxiety, alcohol abuse, somatoform disorder, and eating disorder. It is a self-report questionnaire that assesses the levels of depression on the nine key symptoms (each rated from 0-3) in the past two weeks. The scores on the questionnaire range from 0 to 27: a score of 10 or higher is indicative of moderate or severe depression and is used to consider major depressive disorder present[3,5-8]. The score can also be used as a measure of depression severity[3,9]. A categorical algorithm has also been developed to determine major depressive disorder with the PHQ-9[3,9]. The PHQ-2 includes the first two items of the PHQ-9, 'any depressed feelings' and 'any loss of interest'[10] and ranges from 0 to 6. In order to detect major depressive disorder with the PHQ-2, a threshold of 3 is recommended.

Several studies validated the performance of both questionnaires in a variety of patient populations, most of them showing good accuracy[3,5-8,11-16]. However, the PHQ-9 has not yet been validated in primary care in the Netherlands. Furthermore, very few studies validated the accuracy of the PHQ-2[10,12,16,17].

We validated both the PHQ-9 and the PHQ-2 in a large Dutch primary care patient cohort addressing three questions: (1) Is the PHQ-9 a reliable and valid measurement of major depressive disorder in primary care? Reliability refers to internal consistency as well as test-retest reliability. Validity refers to construct validity, i.e. is the PHQ-9 an adequate measurement of depression severity; 2) Does the threshold score of 10 and the categorical algorithm for the PHQ-9 yield accurate classification in primary care?; (3) What is the accuracy of the PHQ-2 for major depressive disorder in primary care?

## Methods

### Patients and design

We used patient data of the PREDICT-NL study, which is the Dutch part of the PredictD study. The design and primary results of the PredictD study have been published previously[18,19]. In brief, PredictD is a large prospective cohort study that started in 2003 from which a multifactor risk algorithm was developed for the onset of major depression over 12 months in primary care in 6 European countries and Chile[18]. Consecutive general practice patients were asked to participate, irrespective of their reasons for consulting the general practitioner. The study was approved by the Medical Ethics committee of the universities of participating countries.

In the Netherlands, patients were recruited from seven general practices in the city of Utrecht and surrounding areas. On random days, research assistants visited the general practices to recruit patients. Patients aged 18 years or older who visited the general practice were asked to participate while waiting to see the general practitioner. Patients interested in participating were given oral and written information about the study aims and procedure. If patients were willing to participate, they received the study information sheet, an informed consent form, and the questionnaires. The patient was asked to take the material home, read the study information and ask for additional information if necessary. After having signed the informed consent form they filled out the questionnaire and returned the signed informed consent form and questionnaire by regular mail. Nonresponders were sent a reminder after two weeks and again after four weeks.

To assess the test-retest reliability of the PHQ-9, thirty-two consecutively included study participants in one general practice were asked to fill out the PHQ questionnaire for a second time after 14 days.

### Diagnosis of major depressive disorder (reference standard)

The diagnosis of major depressive disorder was assessed in all patients according to DSM-IV criteria[20] by trained researchers using the depression section of the Composite International Diagnostic Interview (CIDI)[21]. When informed consent and the questionnaire were received, the researchers phoned the participant and asked the two core questions of the depression section of the CIDI interview[21], i.e. did you have a depressed mood or a loss of interest for a 2-week-period or longer in the past six months. If the participant responded negative to both questions a diagnosis of major depressive disorder was ruled out[20,21]. If the participant responded positive on one or both questions, an appointment was made in the general practice to conduct the full CIDI depression interview to establish the presence of major depressive disorder. If the participant was unable to schedule the interview at the general practice, the interview was done by telephone (26% of the interviews). The electronic processing of the questionnaires was done completely separate from the CIDI interview, thus effectively blinding the researchers from the PHQ-9 answers.

### Patient health questionnaire

Each of the nine questions of the PHQ-9 was evaluated on a 4-point rating scale, ranging from 0 (not at all) to 3 (nearly every day), summing up to a total PHQ-9 score per patient. Major depressive disorder was considered present if the score was >= 10[3,5-8]. For the categorical algorithm, the answers on the questions were dichotomized: 0 (not at all) and 1 (several days) are coded as 0 (symptom absent) and the answers 2 (more than half the days) and 3 (nearly every day) are coded as 1 (symptom present). The diagnosis of major depressive disorder is made when at least five symptoms are present, and at least one is 'depressed feelings' or 'loss of interest'[3,20]. For the PREDICT-NL study, the Dutch version of the PHQ-9 was developed using several steps of translating and back-translating by researchers and professional translators, one of whom was a native English speaker. The PHQ-2 is a reduced version of the PHQ-9: only the core symptoms of major depressive disorder ('depressed feelings' and 'loss of interest'), the first two items, are measured as described above, summing up to a total that ranges from 0 to 6.

### Functional status, sick days, and number of consultations

We also assessed other parameters to evaluate the validity of the PHQ-9. These were:

1) Functional status using the Medical Outcome Study Short Form General Health Questionnaire-12 (SF-12)[22]. This instrument is divided into scales for mental and physical health, where higher scores indicate better functioning.

2) Information on the number of days in the past 4 weeks that patients were unable to perform usual activities due to health problems (number of sick days).

3) The number of general practice consultations in the past 12 months was counted using the electronic database of the general practitioners. This was assessed as a measure of health service utilisation.

### Data analysis

We estimated the internal consistency, the degree to which the answers on the individual questions of the PHQ-9 are the same, using intraclass correlations and the test-retest correlation were estimated using Pearson correlations. To assess the validity of the PHQ-9 as a measurement of depression severity, scores were divided in categories of increasing severity: 0-4, 5-9, 10-14, 15-19 and 20 and higher, as used in other studies[3]. Medians and interquartile ranges of the functional status (SF-12), sick days and the number of consultations in the previous 12 months were estimated across these categories. Differences between categories were tested with Kruskal-Wallis analysis of variance. Differences in PHQ-9 and PHQ-2 scores between patients with and without major depressive disorder were tested with the Mann-Whitney U test. P-values of 0.05 and lower were considered significant.

We then estimated the concordance-statistic (c-statistic or area under the Receiver Operating Characteristic curve) for the PHQ-9. The sensitivity, specificity, and positive and negative predictive value were estimated for several thresholds of the PHQ-9 overall score and for the categorical algorithm of the PHQ-9. Finally, the c-statistic was constructed for the PHQ-2 and sensitivity, specificity, positive and negative predictive value were calculated for all possible thresholds of the PHQ-2.

The overall percentage of missing values was 9%. As missing data rarely occur at random, it is widely acknowledged that simple deletion of patients with one or more missing values (i.e. complete case analysis) leads to biased results[23-26]. We therefore used single imputation to address missing values. The imputation and analysis was done in SPSS version 15 (SPSS inc. Chicago, Ill).

## Results

**Figure 1:**
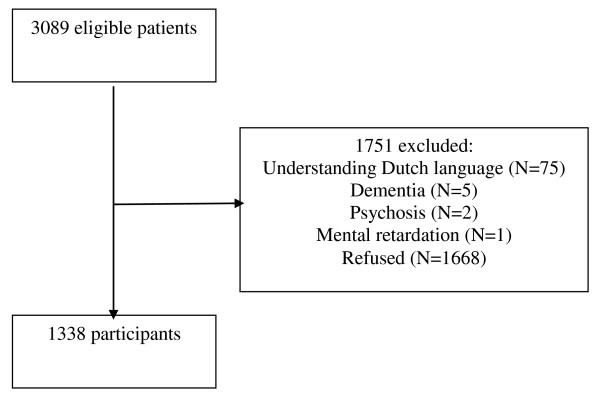
**Flow chart of the inclusion of patients**.

In total, 3089 patients were asked to take part in the PREDICT-NL study, 83 of whom did not meet inclusion criteria, mainly (n = 75) because they had problems understanding the Dutch language. An additional 8 patients were excluded because the general practitioner confirmed that they had dementia (n = 5), psychosis (n = 2), or mental retardation (n = 1). Of the 3006 eligible patients, 1338 (44.5%) gave written informed consent and participated in the study (Figure 1). Reasons for not participating were mostly lack of time and no interest in the study.

The mean age of the study population was 51 years (SD = 16.7), and the majority (63%) was female (Table 1). Thirty five patients (2.6%) consulted the general practitioner for mood related health problems. DSM IV Major depressive disorder according to the CIDI was diagnosed in 176 (13%) patients and was more prevalent in women and younger patients (Table 1). Patients with major depressive disorder had significantly higher scores on the PHQ-9 (p < .000) and PHQ-2 (p < .000) compared with patients without depression.

**Table 1:** Distribution of patient characteristics according to diagnostic status for major depressive disorder

	Major depressive disorder		Total
	No (N = 1176)	Yes (N = 176)	
Male gender, n (%)	448 (39)	50(28)	498 (37)
Age (years), mean(SD)	52 (17)	46(14)	51 (17)
PHQ-9, median(IQR^1^)	2 (1-5)	9 (6-14)	3 (1-6)
PHQ-2, median(IQR)	0 (0-1)	2 (2-4)	0 (0-2)
Physical functioning, SF-12, median(IQR)	50 (40-54)	48 (40-57)	49 (40-54)
Mental functioning, SF-12, median(IQR)	52 (45-56)	30 (25-38)	50 (41-56)
Sick days (n), median(IQR)	0 (0-5)	3 (0-10)	0 (0-5)
Consultations in previous 12 months(n), median(IQR)	9 (5-16)	12 (7-20)	10 (5-16)

^1 ^IQR - interquartile ranges

Thirty-one of the 32 patients approached agreed to fill in the PHQ-9 for a second time. The association between the test and retest scores was excellent, with a correlation of 0.94. The internal consistency of the PHQ-9 was very good with an intraclass correlation of 0.88.

In table 2 the medians of the SF12, the number of sick days and the number of consultations are shown for patients in different PHQ-9 categories. A statistically significant difference in quality of life was observed for patients with different levels of depressive symptoms, with patients with higher levels of depression reporting a lower quality of life. This difference was more pronounced on the mental functioning than the physical functional scale. A statistical significant difference was also observed on the reported number of sick days in the past 4 weeks and number of consultations in the past twelve months (all p-values < 0.001).

**Table 2:** Association between PHQ-9 depression score and SF-12 health related quality of life scores, sick days and number of consultations in the past 12 months.

	Level of depression severity (PHQ-9 score)					
	Minimal (0-4)	Mild (5-9)	Moderate (10-14)	Moderately severe (15-19)	Severe (20-27)	**p-value**^**2**^
Physical functioning, Median (IQR^1^)	50 (42-54)	47 (38-54)	47 (38-57)	41 (34-52)	42 (39-50)	0.00
Mental functioning, Median (IQR)	54 (49-57)	42 (33-49)	30 (25-36)	26 (21-34)	21 (17-28)	0.00
Sick days, median (IQR)	0 (0-4)	1 (0-7)	6 (0-15)	7 (2-20)	8 (1-15)	0.00
Consultations in previous 12 months, Median (IQR)	9 (5-15)	11 (6-17)	14 (8-22)	16 (7-27)	12 (9-25)	0.00

^1^IQR - Interquartile range

^2 ^P-values from Kruskal-Wallis tests

The area under the ROC curve of the PHQ-9 was 0.87 (95% CI: 0.84-0.90). Table 3 shows the accuracy measures for different thresholds of the PHQ-9 score. The commonly used threshold of 10 had a specificity of 0.95 but a sensitivity of only 0.49. At a threshold of 6, sensitivity was 0.82 and specificity was 0.82. At this threshold the a-priori probability (prevalence) of 13% was increased to a posterior probability of 41%.

**Table 3:** Sensitivity, specificity, and predictive values for different thresholds of the PHQ-9 and the PHQ-2.

PHQ-9 threshold	Sensitivity (95% CI)	Specificity (95% CI)	PPV (95% CI)	NPV (95% CI)
≥4	0.89 (0.84-0.94)	0.64 (0.61-0.67)	0.27 (0.23-0.31)	0.97 (0.96-0.98)
≥5	0.86 (0.81-0.91)	0.75 (0.73-0.77)	0.34 (0.30-0.38)	0.97 (0.96-0.98)
≥6	0.82 (0.76-0.88)	0.82 (0.80-0.84)	0.41 (0.36-0.46)	0.97 (0.96-0.98)
≥7	0.74 (0.68-0.80)	0.87 (0.85-0.89)	0.47 (0.41-0.53)	0.96 (0.95-0.97)
≥8	0.65 (0.58-0.72)	0.90 (0.88-0.92)	0.51 (0.44-0.58)	0.94 (0.93-0.95)
≥9	0.57 (0.50-0.64)	0.93 (0.92-0.94)	0.55 (0.48-0.62)	0.94 (0.93-0.95)
≥10	0.49 (0.42-0.56)	0.95 (0.94-0.96)	0.59 (0.51-0.67)	0.93 (0.92-0.94)
≥11	0.44 (0.37-0.51)	0.96 (0.95-0.97)	0.61 (0.53-0.61)	0.92 (0.90-0.94)
≥12	0.39 (0.32-0.46)	0.97 (0.96-0.98)	0.64 (0.55-0.74)	0.92 (0.89-0.93)

**PHQ-9 algorithm**	0.28 (0.21-0.35)	0.98 (0.97-0.99)	0.69 (0.58-0.80)	0.90 (0.88-0.92)
**PHQ-9 adjusted algorithm**	0.84 (0.79-0.89)	0.81 (0.79-0.83)	0.40 (0.35-0.45)	0.97 (0.96-0.98)

**PHQ-2 threshold**				
≥1	0.90 (0.86-0.94)	0.58 (0.55-0.61)	0.24 (0.21-0.27)	0.98 (0.97-0.99)
≥2	0.81 (0.75-0.84)	0.76 (0.74-0.78)	0.34 (0.29-0.39)	0.96 (0.95-0.97)
≥3	0.42 (0.35-0.49)	0.94 (0.93-0.95)	0.53 (0.45-0.61)	0.91 (0.89-0.93)
≥4	0.31 (0.24-0.38)	0.97 (0.96-0.98)	0.64 (0.54-0.74)	0.90 (0.88-0.92)
≥5	0.19 (0.13-0.25)	0.99 (0.98-1.00)	0.69 (0.56-0.82)	0.89 (0.87-0.91)
≥6	0.14 (0.09-0.19)	0.99 (0.98-1.00)	0.67 (0.52-0.82)	0.88 (0.86-0.90)

For details see text.

PPV: Positive predictive value

NPV: Negative predictive value

95% CI: 95% Confidence Interval

The categorical algorithm of the PHQ-9 showed a specificity of 0.98 and sensitivity of only 0.28. Based on this we defined an adjusted categorical algorithm to include the responses 'several days' as symptom present (see methods), whereas the original algorithm codes these answers as symptom absent. This resulted in a sensitivity of 0.84 and specificity of 0.81, close to those found for a threshold of 6 (Table 3). As the time delay between the PHQ-9 and the reference test varied, we performed an additional analysis to determine the influence of this time delay. Discrimination (area under the ROC curve) was similar when the delay between PHQ-9 and CIDI was longer (results not shown).

The area under the ROC curve of the PHQ-2 was 0.83 (95% CI 0.80-0.87). The commonly used threshold for the PHQ-2 of 3 showed a specificity of 0.94 and sensitivity of 0.42 (Table 3). As with the PHQ-9, lower thresholds showed more balanced values of sensitivity and specificity, notably at a threshold of 2. At this threshold, the a-priori probability (prevalence) of 13% was increased to a posterior probability of 34%.

## Discussion

The PHQ-9 showed a very good internal consistency and test-retest reliability. Moreover, more severe depressive symptoms as measured by the PHQ were associated with poorer functional status, sick days, and higher number of general practice consultations. The accuracy of detecting major depressive disorder at the recommended threshold of 10 and for the categorical algorithm, however, was poor. Lowering the threshold and minor adjustments of the categorical algorithm showed a considerable improvement of sensitivity, at the cost of lower specificity (Table 3). The adjusted categorical algorithm included all responses other than 'Not at all' as item present. The PHQ-2 showed a similar level of accuracy (i.e. sensitivity and specificity) when a lower threshold of 2 rather than 3 was used.

Our results of the reliability and construct validity of the PHQ-9 are similar to those reported in another primary care study[3] and a study of chronically ill primary care patients[13]. When we compared our observed sensitivities and specificities with other studies, we noted mixed results in the existing literature. A systematic review of the PHQ-9 in primary care found a pooled sensitivity of 0.77 (95% CI: 0.71-0.84) and a pooled specificity of 0.94 (95% CI: 0.90-0.97) for the diagnostic algorithm [15]. Similar results were found for the threshold of 10 in a systematic review by Gilbody et. al. [12]. Both reviews report substantially higher sensitivities compared to those reported here. However, a number of other studies in specific patients populations (e.g. patients with cardiovascular diseases) also observed low sensitivities and comparable specificities as we observed[13,17,27]. Similarly, the recommended threshold of 3 for the PHQ-2 showed a low sensitivity in comparison with other primary care studies[7,10,28], whereas other studies describe results similar to those reported here[16,17,29].

Strengths of this study are, first, that patients were included consecutively on random days, irrespective of their presented symptoms or signs and thus representing all patients in the waiting room of the GP. Second, patients were approached for participation in several general practices in both rural and urban areas to ensure a representative sample. Third, the reference test was administered by well-trained CIDI interviewers to guarantee the validity of the diagnoses and was applied in all attendees so that there was no selection bias. Fourth, this is the first study that validates the PHQ-9 and PHQ-2 in Dutch primary care.

Our study also has some limitations. First, the non-response rate for this study was relatively high. However, we found very minor non-significant differences in distributions of gender and age compared to responders (data not shown). Second, the prevalence of major depressive disorder in this study was relatively high[30-32]. It is possible that patients with major depressive disorder or similar mood problems were more willing to participate in our study. As a result, we would expect sensitivities and positive predicted values to be overestimated and specificities and negative predictive values to be underestimated[33]. This, however, is not consistent with the results presented here and therefore unlikely to explain our findings. Third, the test-retest reliability was assessed in only 31 patients. Still, the results were very similar to earlier findings[3,13]. Fourth, the questionnaire was filled out at home. It is therefore possible that the answers were influenced by others (e.g. family members). However, this influence had to be systematically in one direction for patients with major depressive disorder and more or less absent for all other patients to explain our findings, which is unlikely. Furthermore, there was a time delay between the PHQ-9 and the CIDI. However, in an additional analysis, we observed no influence of the time delay on sensitivities and specificities of the PHQ-9. Also, a substantial part of the CIDI interviews was administered by telephone. Previous studies, however, have shown that telephone interviews are valid for clinical assessment of depression [31,34]. It has been suggested that the CIDI underdetects major depressive disorder when compared to the SCID [31]. In larger clinical or epidemiological studies, however, it is not feasible to administer the SCID in all patients because this is a semi-structured interview that has to be administered by clinicians instead of trained lay-persons. Also, most critical evaluations of the CIDI were based on earlier versions than the version (2.1) used in our study[35].

The limitations of our study cannot, in our view, explain the low sensitivities for detecting major depressive disorder we observed. Differences between the PHQ-9 and reference tests such as the CIDI and the SCID, have been previously described[15]. The PHQ-9 is designed to inquire about symptoms of major depressive disorder in the past 2 weeks rather than the past 12 months (adapted to the past 6 months in our study) for the CIDI. Patients with symptoms of major depressive disorder in the past 6 months and less severe symptoms in the past 2 weeks will not be detected with the PHQ-9 or the PHQ-2. Conversely, patients reporting little or no symptoms in the CIDI interview will also report no symptoms on the PHQ-9. As such, this difference in time frame could very easily result in low sensitivities and high specificities for the PHQ-9 threshold and algorithm and the recommended threshold for the PHQ-2.

The currently recommended high thresholds will lead to large numbers of undetected depressions. Before applied in clinical practice, lower threshold values as considered in the present study should be evaluated in other studies with new patients and different settings. The high negative predictive value and a relative low positive predictive value at the lower threshold of 6 (Table 3) showed that exclusion of major depressive disorder is more feasible than inclusion. Even though the positive predicted value of 41% still represents a considerable increase of the a-priori probability of 13%, it also emphasizes the need for a further diagnostic work-up for major depressive disorder in patients with a high score on the PHQ-9.

## Conclusion

In conclusion, the results presented here indicate that the PHQ-9 and the PHQ-2 are useful instruments to detect major depressive disorder in primary care. As the positive predictive value is still low, a high score needs to be followed by an additional diagnostic work-up. In addition, the PHQ-9 is a valid measurement of depression severity. For both scales, however, clinicians should be aware that current recommended thresholds could lead to under detection.

## Competing interests

The authors declare that they have no competing interests.

## Authors' contributions

MK and IN originated the idea for the Predict study and led on its design. MIG and MJvW supervised and participated in the data collection in the Netherlands. NPAZ, YV, MJvW, KGMM and MIG originated the idea for this paper, analysed the data, interpreted the results and wrote and revised the paper. MK and IN revised the manuscript, All authors read and approved the final manuscript.

## Pre-publication history

The pre-publication history for this paper can be accessed here:

http://www.biomedcentral.com/1471-2296/11/98/prepub
